# Supplementary material for: An oxidative metabolic pathway of 4-deoxy-L-erythro-5-hexoseulose uronic acid (DEHU) from alginate in an alginate-assimilating bacterium
Source: Commun Biol. 2021 Nov 2;4:1254. doi: 10.1038/s42003-021-02786-8 (PMC8563752; doi:10.1038/s42003-021-02786-8)
Supplement: Supplementary file 2 — Supplementary information. [file 42003_2021_2786_MOESM2_ESM.pdf]

Supplementary Information

An oxidative metabolic pathway of 4-deoxy-L-*erythro*-5-hexoseulose uronic acid (DEHU) from alginate in an alginate-assimilating bacterium

Ryuji Nishiyama[1], Takao Ojima[1], Yuki Ohnishi[2], Yasuhiro Kumaki[3], Tomoyasu Aizawa[2], and Akira Inoue[1]

[1] Graduate School of Fisheries Sciences, Hokkaido University, Hakodate, Hokkaido, Japan

[2] Faculty of Advanced Life Science, Hokkaido University, Sapporo, Hokkaido, Japan

[3] Faculty of Sciences, Hokkaido University, Sapporo, Hokkaido, Japan

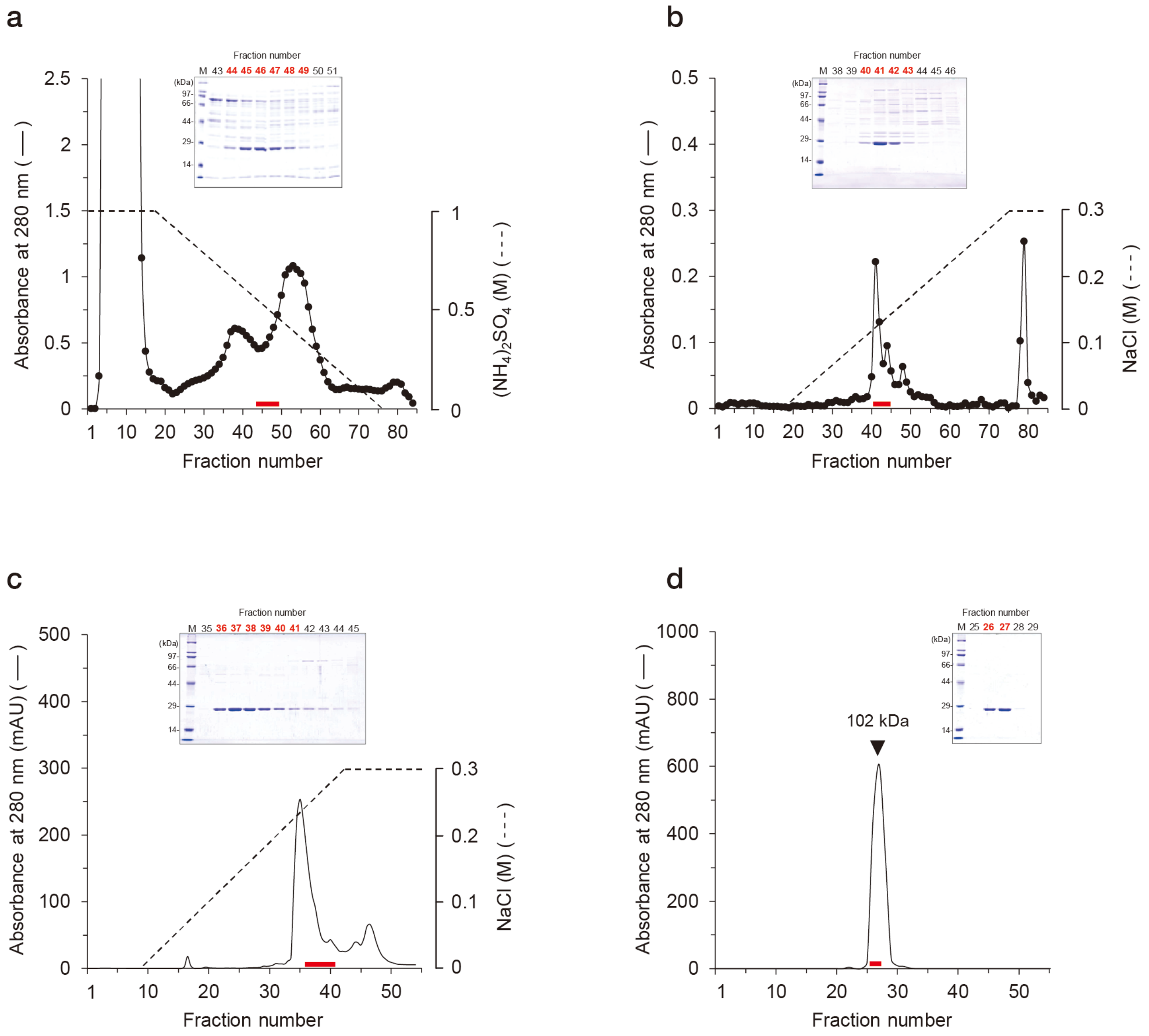

Supplementary Fig. 1. Purification of the enzyme possessing DEHU-oxidizing activity from strain UMI-01.

An enzyme with DEHU-oxidizing activity was purified from the cell lysate of strain UMI-01 using sequential column chromatography on TOYOPEARL Butyl-650M (**a**), TOYOPEARL SuperQ-650S (**b**), MonoQ 4.6/100 PE (**c**), and Superdex 200 10/300 GL (**d**). The active fractions were detected using the TLC assay and the fractions indicated by red lines were subjected to the next round of chromatography (**a–c**) or were pooled (**d**). Closed triangle in **d** indicates elution point of the target enzyme, and its molecular weight was estimated to be 102 kDa. *mAU*, milli absorbance unit.

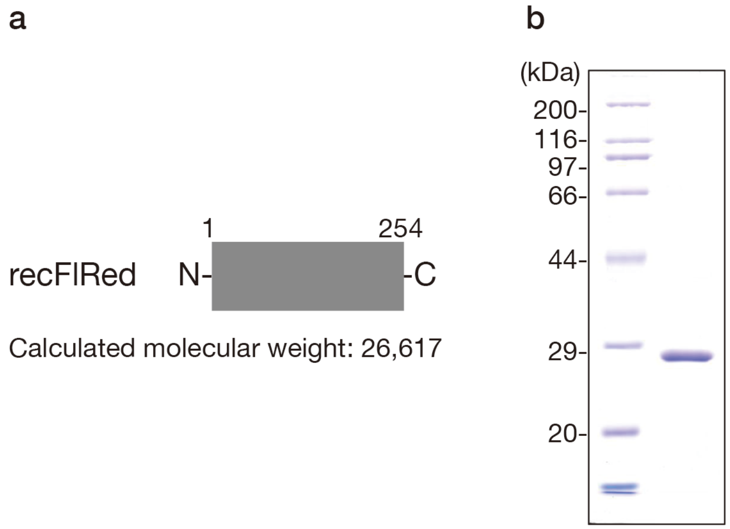

Supplementary Fig. 2. Schematic drawing and SDS-PAGE of recFlRed.

a, Schematic diagram of recFlRed. **b**, SDS-PAGE of purified recFlRed. Left and right lanes represent protein marker and purified recFlRed, respectively.

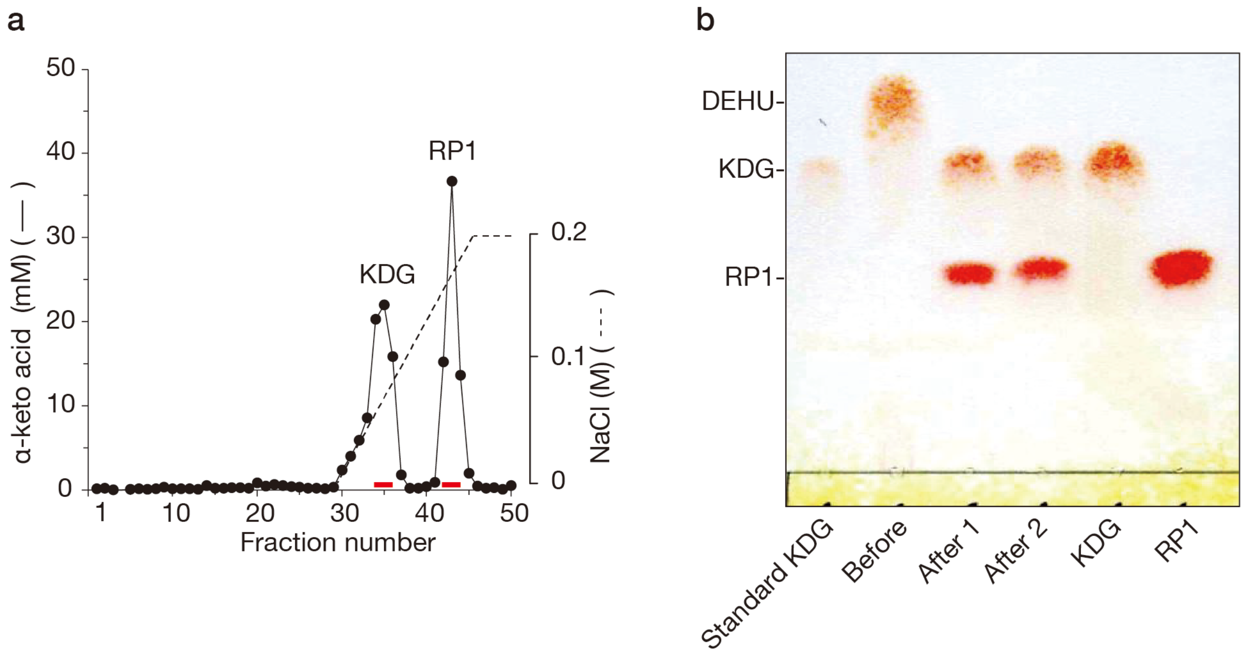

Supplementary Fig. 3. Purification of the reaction products of recFlRed.

a, Column chromatography of reaction mixture of DEHU and recFlRed on TOYOPEARL SuperQ-650. **b**, TLC analysis of DEHU and two fractions indicated as "KDG" and "RP1" in **a**. *Standard KDG*, commercially available KDG; *Before*, before enzyme reaction; *After 1*, sample after enzyme reaction; *After 2*, dissolved precipitates after 2-propanol treatment of "After 1"; *KDG* and *RP1*, fractions indicated by red lines in **a**, respectively.

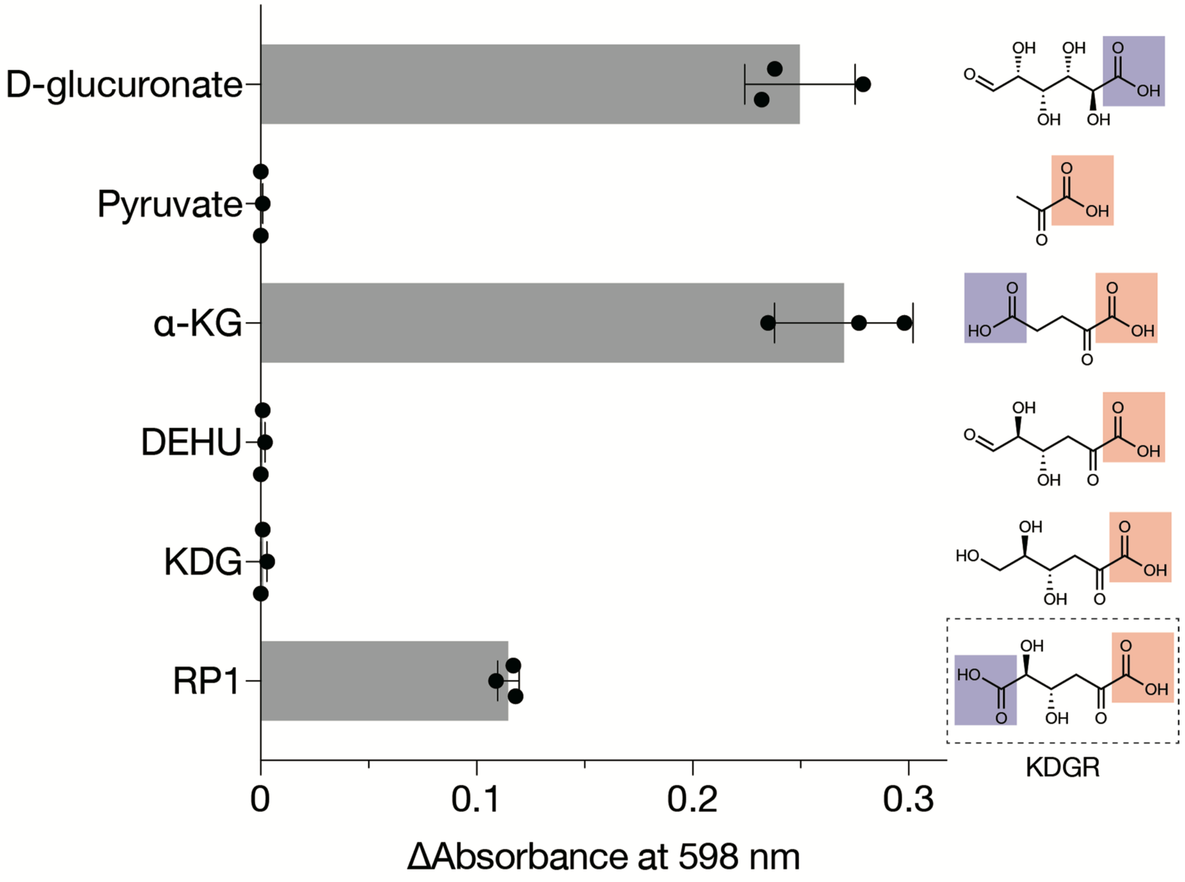

Supplementary Fig. 4. Detection of carboxyl groups using 4-nitrophynacyl bromide.

The structures of the examined compounds (D-glucuronate, pyruvate, α-KG, DEHU, and KDG) and the predicted structure (KDGR) of RP1 are shown. The carboxyl groups in the α-keto acid structures and the other carboxyl groups are highlighted in red and blue, respectively. Values of Δabsorbance were obtained by subtracting the absorbance of each solution containing the indicated compound from that of the buffer. All assays were repeated thrice and the data are shown as mean ± S.D.

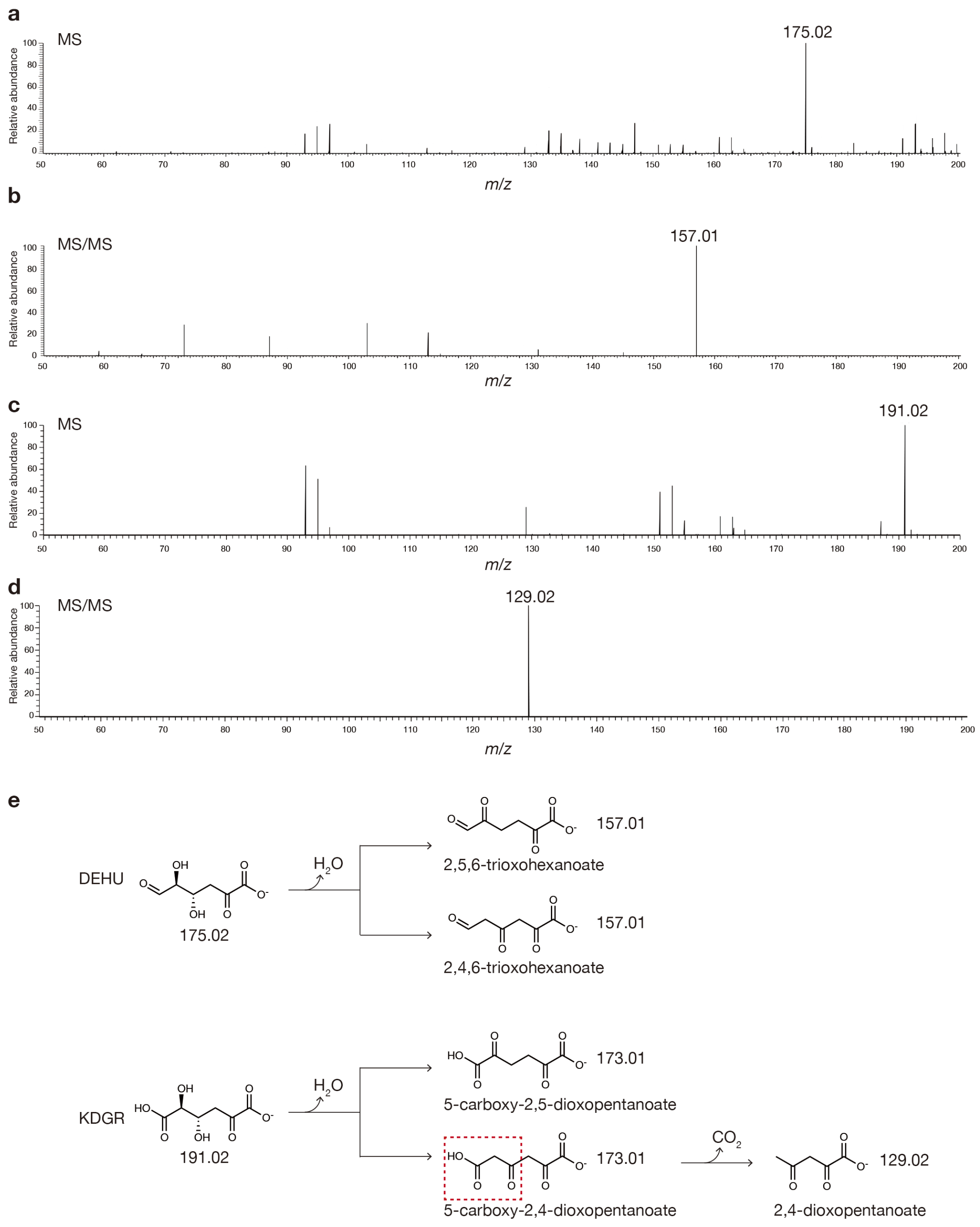

Supplementary Fig. 5. ESI-MS and MS/MS spectra of DEHU and RP1.

a, ESI-MS spectrum of DEHU. **b**, MS/MS spectrum of the peak at *m/z* 175.02 in **a**. **c**, ESI-MS spectrum of RP1. **d**, MS/MS spectrum of the peak at *m/z* 191.02 in **c**. **e**, Possible fragmented structures of DEHU and KDGR. The red-dotted square indicates the β-keto acid structure. Each calculated molecular mass is shown.

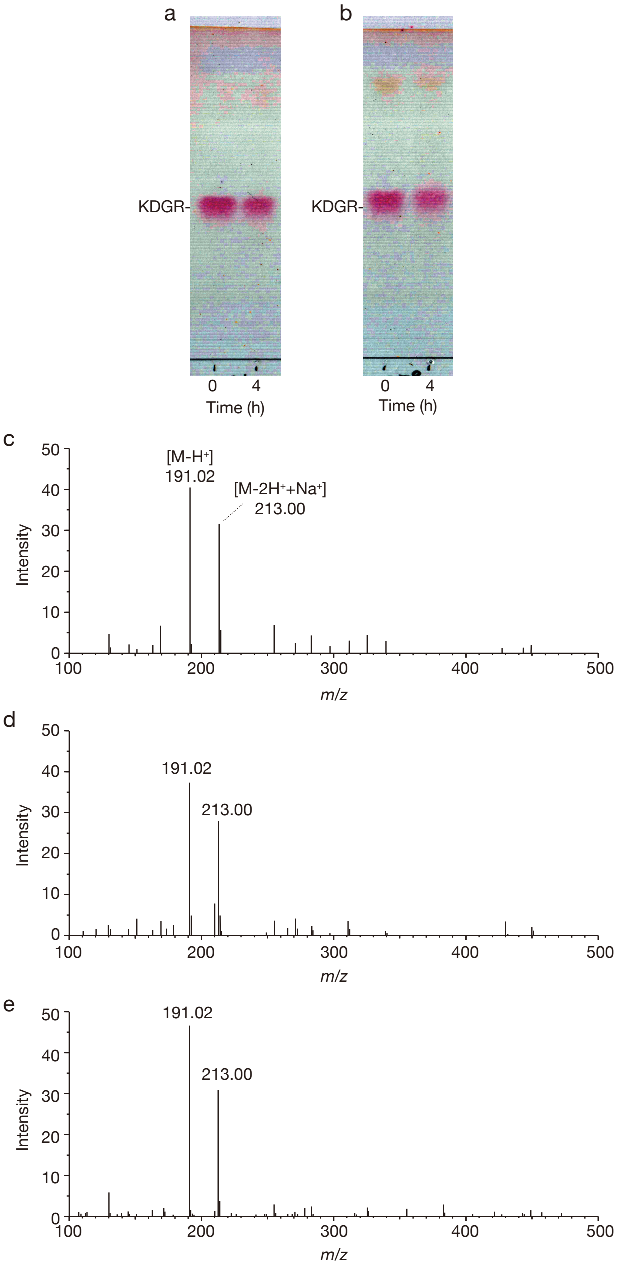

Supplementary Fig. 6. Reaction test of FlKin or FlAld to KDGR.

Recombinant FlKin (recFlKin) and FlAld (recFlAld) were prepared as described previously[1]. Reactions were conducted at 30˚C for 4 h in the mixture 10 mM sodium phosphate (pH 7.4), 100 mM NaCl, 4 mM $MgCl_2$, 2 mM ATP, 2 mM KDGR, and 20 µg mL^{-1} recFlKin or the mixture 10 mM sodium phosphate (pH 7.4), 100 mM NaCl, 2.5 mM KDGR, and 10 µg mL^{-1} recFlAld. *a* and *b*, TLC analysis (TBA staining method) of reaction mixtures containing recFlKin and recFlAld, respectively. *c*, *d*, and *e*, ESI-MS spectrum of KDGR before reaction, the products after recFlKin reaction, and after recFlAld reaction, respectively.

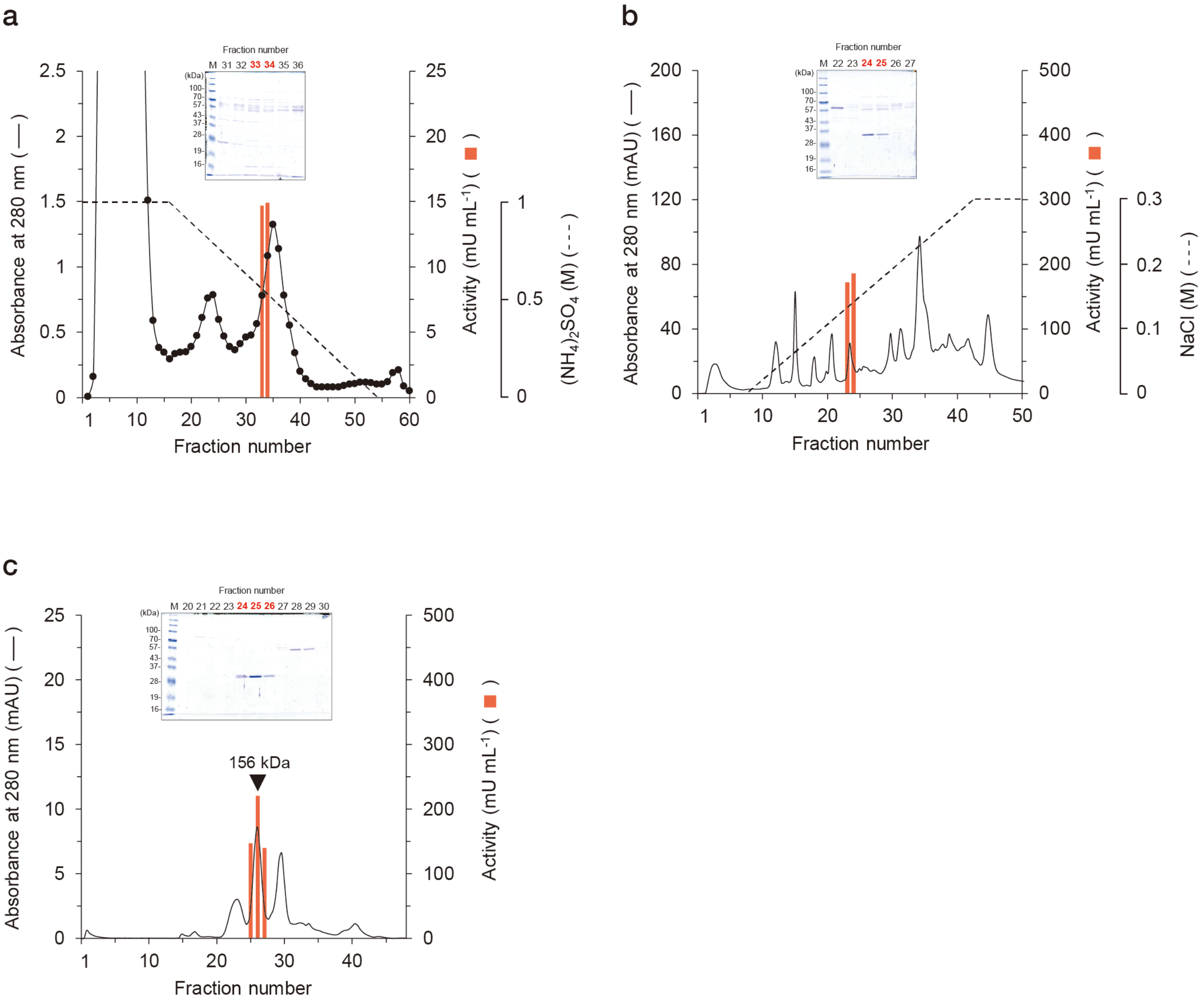

Supplementary Fig. 7. Purification of an enzyme catalyzing KDGR as a substrate from strain UMI-01.

The target enzyme was purified from cell lysate of strain UMI-01 using sequential column chromatography on TOYOPEARL Butyl-650M (**a**), MonoQ 4.6/100 PE (**b**), and Superdex 200 10/300 GL (**c**). The active fractions were detected using the TBA method, and the fractions indicated by red lines were subjected to next round of chromatography (**a**,**b**) or were pooled (**c**). *mAU*, milli absorbance unit.

```
FlDet-F
ccagattggtttaccagtttggatttccaaatataggtatatttattgatttacataacatttgtttaaaaaaaaaataa  80
aaATGACAACACTATCAGCTTCTACAAAAGAGAAATTAAAAACAGTGAGCACGCCCACAATTGCTACATGTTTGTATAAA 160
  M  T  T  L  S  A  S  T  K  E  K  L  K  T  V  S  T  P  T  I  A  T  C  L  Y  K   26
AAAGGGTTTAAAAATCAATTTATTCAAGATGTTAAACCTTTGCAATTAGGCAAACCGACCATGGTGGGGGAGGCCTTTAC 240
 K  G  F  K  N  Q  F  I  Q  D  V  K  P  L  Q  L  G  K  P  T  M  V  G  E  A  F  T  53
CTTGCGCTATATTCCAGCTAGAGAAGATCGTAATCCACTGACTGTTTTTAGAAATGCCGATCATCCACAACGTGTAGCAA 320
  L  R  Y  I  P  A  R  E  D  R  N  P  L  T  V  F  R  N  A  D  H  P  Q  R  V  A   79
TCGAAAGTTGTCCTGTAGGATGTGTGTTGGTAATGGATAGTCGTAAAGATCCTCGTGCTGCCTCGGCTGGGGATATTTTG 400
I  E  S  C  P  V  G  C  V  L  V  M  D  S  R  K  D  P  R  A  A  S  A  G  D  I  L 106
GTAACGCGACTTATGGTGCGGGGAGCTGCTGGTATTGTGACCGATGGTGGTTTCAGGGATTCGGCATCCATTGCTAAACT 480
 V  T  R  L  M  V  R  G  A  A  G  I  V  T  D  G  G  F  R  D  S  A  S  I  A  K  L 135
GCCTTTTCCATCGTATCATAATCGTCCATCGGCGCCGACTAATTTAACTTTACATGAAGCTTTGGATATAAATATTCCTA 560
  P  F  P  S  Y  H  N  R  P  S  A  P  T  N  L  T  L  H  E  A  L  D  I  N  I  P  159
TTGGCTGCGGTGATGTGGCTGTTTTTCCAGGTGATGTAGTAGTAGGTGATGATGATGGAGTCATTGTAATTCCTGCTCAT 640
I  G  C  G  D  V  A  V  F  P  G  D  V  V  V  G  D  D  D  G  V  I  V  I  P  A  H 186
ATTGCTGATGAAGTTGCTGCAGAATGTGTAGAAATGACGCTATATGAAAATTTTGTTTTAGAAAAAGTGGCTCAGGGAAG 720
 I  A  D  E  V  A  A  E  C  V  E  M  T  L  Y  E  N  F  V  L  E  K  V  A  Q  G  S 213
TACGATTATTGGGCTTTATCCTCCAACTAACGAAGAGAATTTAGTAGCGTTTGAAAACTGGAAAAAAAATAAATAGatga 800
  T  I  I  G  L  Y  P  P  T  N  E  E  N  L  V  A  F  E  N  W  K  K  N  K  *     237
gattgttgaggtaatcaggcgagtgatgggtcactta                                            837
                                                                          FlDet-R
```

Supplementary Fig. 8. DNA and the amino acid sequences of FlDet.

Red letters indicate sequences that match the results of N-terminal sequence analysis of native FlDet. The start and stop codons are underlined. The positions of the primers used for genomic PCR are indicated by arrows.

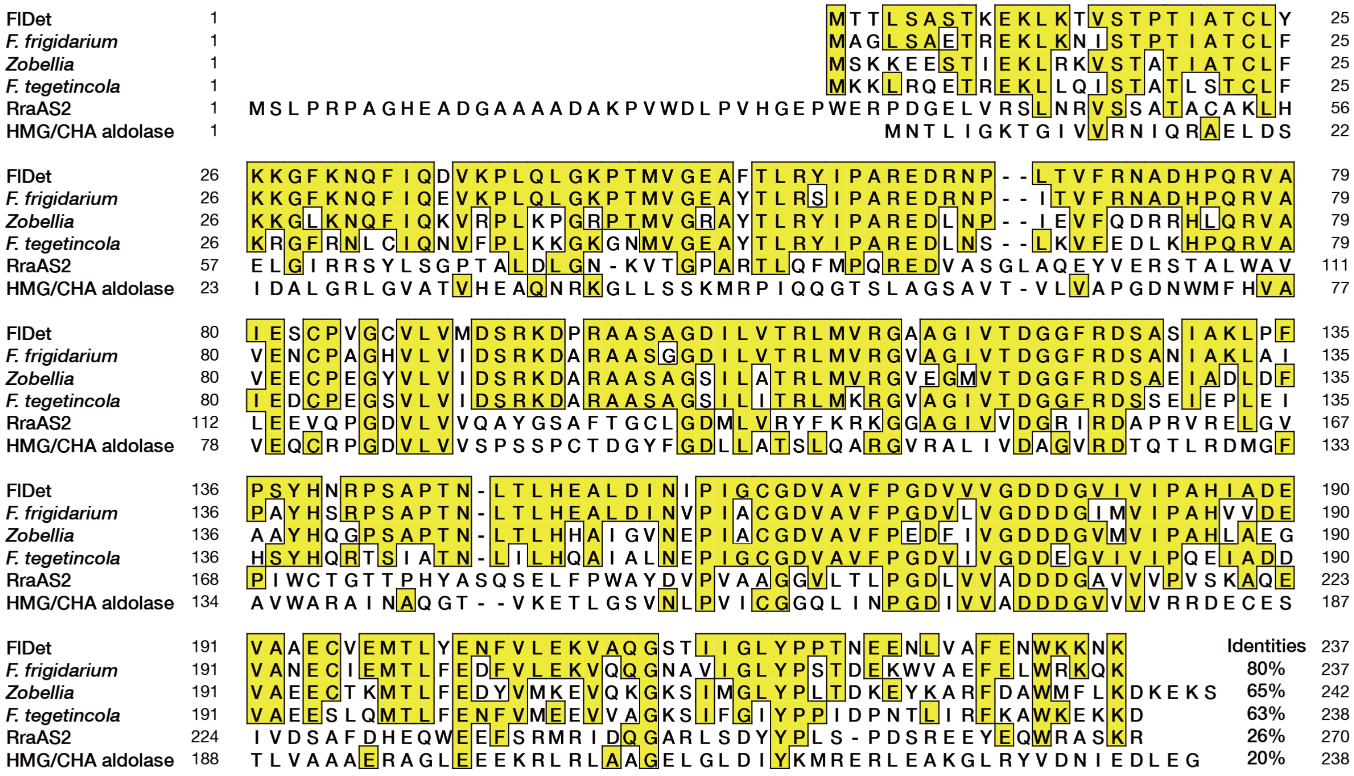

Supplementary Fig. 9. Comparison of the amino acid sequences of FlDet and its homologs.

FlDet, *Flavobacterium sp*. strain UMI-01 FlDet (in this study); *F. frigidarium*, ribonuclease activity regulator RraA-like protein from *Flavobacterium frigidarium* (GenBank accession number WP_026708034); *Zobellia*, ribonuclease activity regulator RraA-like protein from *Zobellia galactanivorans* (GenBank accession number WP_013993973); *F. tegetincola*, ribonuclease activity regulator RraA-like protein from *F. tegetincola* (GenBank accession number WP_026978740); *RraAS2*, RNase E inhibitor from *Streptomyces coelicolor* A3 (GenBank accession number NP_631222); *HMG/CHA aldolase*, 4-hydroxy-4-methyl-2-oxoglutarate (HMG)/4-carboxy-4-hydroxy-2-oxoadipate (CHA) aldolase from *Pseudomonas putida* F1 (GenBank accession number WP_012051536).

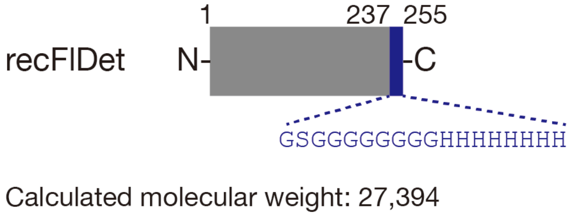

Supplementary Fig. 10. Schematic showing recFlDet.

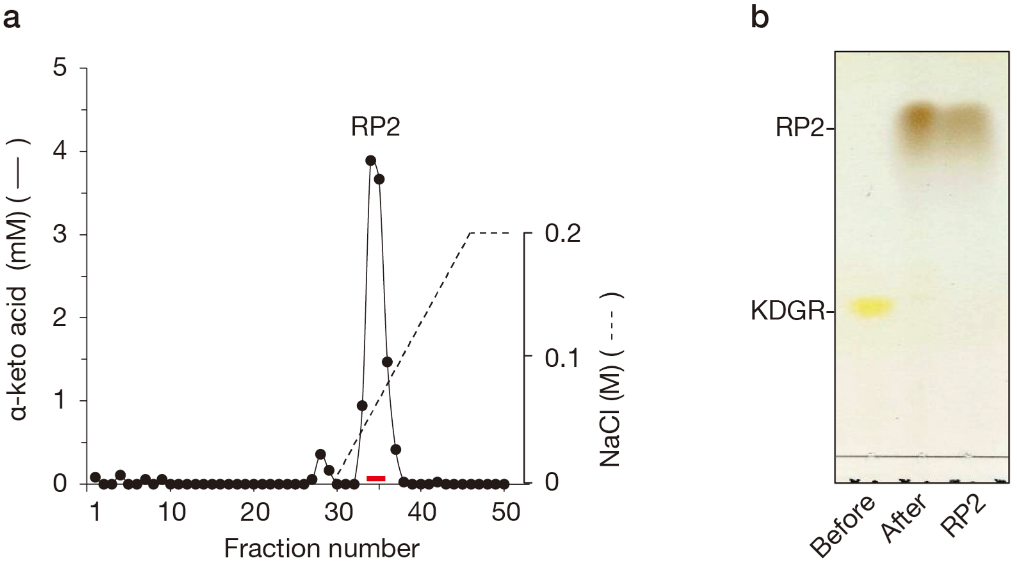

Supplementary Fig. 11. Purification of the reaction products of recFlDet.

a, Column chromatography of the reaction mixture of KDRG and recFlRet on TOYOPEARL SuperQ-650. **b**, TLC analysis of the reaction products of KDRG catalyzed by recFlDet. *Before*, before enzyme reaction; *After*, after enzyme reaction; *RP2*, fractions indicated by redlines in **a**. The spots were visualized by heating at 120°C for 15 min after spraying 2,4-dinitrophenyl hydrazine.

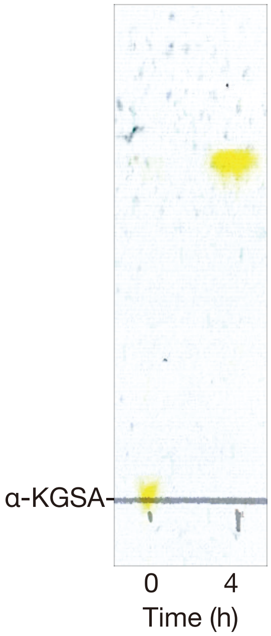

Supplementary Fig. 12. Conversion of α-KGSA **by cell extract from *Flavobacterium* sp. strain UMI-01.**

Reaction was conducted in 10 mM potassium phosphate (pH 7.4), 100 mM KCl, 1 mM $MgCl_2$, 1 mM DTT, 0.5 mM NAD^+, and 0.8 mg mL^{-1} cell extract, and 1 mM α-KGSA at 25˚C. The sample was mixed with one-sixth the volume of 6.3 mM 2,4-dinitrophenyl hydrazine in 3 M HCl and incubated at 50°C for 45 min. The derived products were extracted from the aqueous layer with an equal volume of ethyl acetate and evaporated *in vacuo*. The yellow solids were dissolved in ethyl acetate and subjected to TLC analysis. The samples were developed using the TLC silica gel 60 plate with 1-butanol: ethanol: 0.5 M acetic acid (7:1:2, *v:v:v*).

```
FlDeg-F
ggttaaccagttatggtattccggtggtttttggttatctttgtactccattcaatcgatttaaatccaaaatttgttt   80
taaaataaaataactgatATGAGCACTCCTCAACAATTTCATGCTTTCAATCCTGCAACAAACGAACCTCTTGAAGACGT  160
                  M  S  T  P  Q  Q  F  H  A  F  N  P  A  T  N  E  P  L  E  D  V    21
TTTTGTTACAACCACCACTCAAGAATTAGACAGAGTTGTACATCAAGCAACAATTGCTTTTGAAACCTATAGAAAAAAAG  240
 F  V  T  T  T  T  Q  E  L  D  R  V  V  H  Q  A  T  I  A  F  E  T  Y  R  K  K     47
ACAAAAACGAAATTGCATTATTTTTAGAACAAATTGCCGAAGAAATTATAAATCTAGGCGAGCCTCTCCTAACAAAATGC  320
D  K  N  E  I  A  L  F  L  E  Q  I  A  E  E  I  I  N  L  G  E  P  L  L  T  K  C     74
CATCTTGAAACTGCTTTACCTATTGCAAGATTACAAGGAGAACGCGGCCGAACTGTTGCGCAATTGCAATTGTTTGCTCA  400
 H  L  E  T  A  L  P  I  A  R  L  Q  G  E  R  G  R  T  V  A  Q  L  Q  L  F  A  Q  101
ATTAGTTCGTGAAGGTTCATGGGTAGAGGCAAAAATTGATACGGCGCAACCTAATAGAACTCCTTTACCTAAATCAGACA  480
 L  V  R  E  G  S  W  V  E  A  K  I  D  T  A  Q  P  N  R  T  P  L  P  K  S  D    127
TCCGTCAAATGCTTGTGCCTTTAGGGCCTGTAGCAGTTTTTGGAGCCAGTAATTTTCCGTTAGCGTTTTCAGTAGCGGGT  560
I  R  Q  M  L  V  P  L  G  P  V  A  V  F  G  A  S  N  F  P  L  A  F  S  V  A  G   154
GGGGATACAGCTTCGGCTTTGGCTGCTGGTTGTCCTGTAATTTTCAAAGGGCACCCTGCTCATCCGGGTACATCGGCGAT  640
 G  D  T  A  S  A  L  A  A  G  C  P  V  I  F  K  G  H  P  A  H  P  G  T  S  A  M  181
GGTGGCGACTGCATTTGAGAAGGCGATAGAAAAATGTGGTATGCCCAAAGGAACTTTTGCATTGGTACAAGGTCATACAA  720
 V  A  T  A  F  E  K  A  I  E  K  C  G  M  P  K  G  T  F  A  L  V  Q  G  H  T    207
ATGAGTTAGGAGCTAATTTAGTGCAACATCCAGCCATAAAAGCAGTTGGTTTTACCGGTTCCTTTACTGGAGGAAAAGCT  800
N  E  L  G  A  N  L  V  Q  H  P  A  I  K  A  V  G  F  T  G  S  F  T  G  G  K  A   234
TTATTTGATTTGGCCAATACGAGACCCGAACCTATTCCAGTATATGCCGAAATGGGAAGCACGAACCCTGTTTTTATTTT  880
 L  F  D  L  A  N  T  R  P  E  P  I  P  V  Y  A  E  M  G  S  T  N  P  V  F  I  L  261
ACCCGAAATTTTAAAAGAAAAAGCTACAGCCATTGCCACAGGTATGGCTCAATCAATTGCACAAGGTGTAGGACAGTTTT  960
 P  E  I  L  K  E  K  A  T  A  I  A  T  G  M  A  Q  S  I  A  Q  G  V  G  Q  F    287
GTACTAACCCAGGACTGGCTTTCATCATAAAATCTGAAGAAGCAGAAACGTACTGTAAGGAACTTTGCCAAAAAATAAAT 1040
C  T  N  P  G  L  A  F  I  I  K  S  E  E  A  E  T  Y  C  K  E  L  C  Q  K  I  N   314
GAGACTCCTGCTGGAACAATGTTGACCGAAGGCATTAGCAAAGCCTATCAAAAAAAGATTGCTGTTACTAATGCATTAGC 1120
 E  T  P  A  G  T  M  L  T  E  G  I  S  K  A  Y  Q  K  K  I  A  V  T  N  A  L  A  341
TCCTAATATAGAAATAGCAAAAGGTCAAACCGCCTCAACTGCCAATGCTGCTGTGGCCACGGTTTTTAAAACATCTCTAC 1200
 P  N  I  E  I  A  K  G  Q  T  A  S  T  A  N  A  A  V  A  T  V  F  K  T  S  L    367
AACACTTTCTCGAAAATCCTTTGCTAGCAGAAGAAAATTTTGGTCCCTCTCAGGTTTTGGTCGAAGCACATAACAAAGAA 1280
Q  H  F  L  E  N  P  L  L  A  E  E  N  F  G  P  S  Q  V  L  V  E  A  H  N  K  E   394
GAAATTTTAGAAGCAGCTAAAAACTTAGAAGGCCATTTGACCGCAACTGTTCATGGTACTATGGCTGATTTAGAAAATTA 1360
 E  I  L  E  A  A  K  N  L  E  G  H  L  T  A  T  V  H  G  T  M  A  D  L  E  N  Y  421
CAAAGACTTGGTACGTTTATTAGAATTAAAAGTAGGACGAATAGTGATAAACGGTTTCCCAACAGGCGTTGAAGTTTGCC 1440
 K  D  L  V  R  L  L  E  L  K  V  G  R  I  V  I  N  G  F  P  T  G  V  E  V  C    447
ATGCCATGGTTCATGGTGGTCCCTATCCAGCAACAACTGCCCCACAAAGCACATCAGTAGGAACACAAGCCATTAAACGT 1520
H  A  M  V  H  G  G  P  Y  P  A  T  T  A  P  Q  S  T  S  V  G  T  Q  A  I  K  R   474
TTTGTACGCCCTGTCTGTTTTCAAGACTATCCTTCGTTCTTACTTCCTGAAGCATTAAAAGATGAAAATCCTAACCAAAT 1600
 F  V  R  P  V  C  F  Q  D  Y  P  S  F  L  L  P  E  A  L  K  D  E  N  P  N  Q  I  501
TTGGAGACTTATTGACGGAGAATTTTCAAAGAAAGCTCTCGCTTAAaaaacaggtcacttagttcaaaaaactaagtgac 1680
 W  R  L  I  D  G  E  F  S  K  K  A  L  A  *                                        515
ccatcactcgcctgattacctc                                                          1702
                  FlDeg-R
```

Supplementary Fig. 13. DNA and the amino acid sequences of FlDeg.

The start and stop codons are underlined. The positions of the primers used for genomic PCR are indicated by arrows.

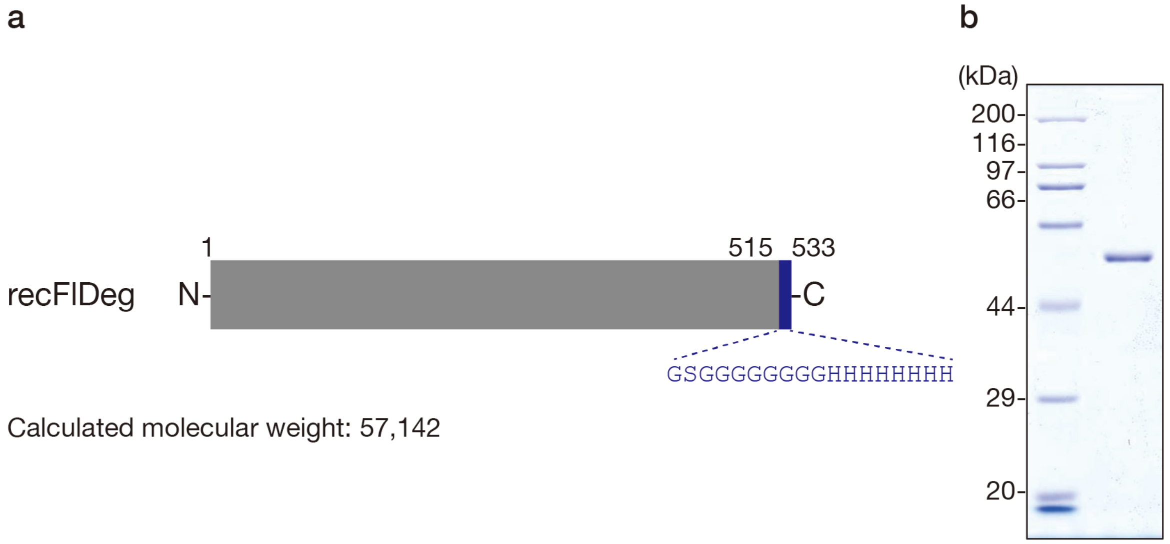

Supplementary Fig. 14. Schematic drawing and SDS-PAGE of recFlDeg.

a, Schematic diagram of recFlDeg. **b**, SDS-PAGE of purified recFlDeg. Left and right lanes represent protein marker and purified recFlDeg, respectively.

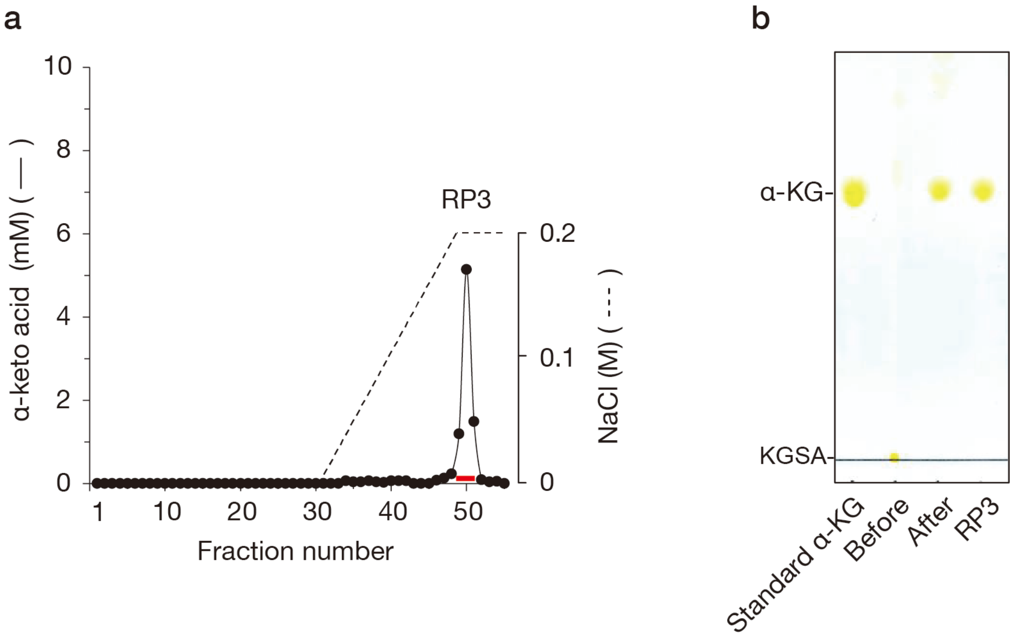

Supplementary Fig. 15. Purification of the reaction products of recFlDeg.

a, Column chromatography of the reaction mixture of α-KGSA and recFlReg on TOYOPEARL SuperQ-650. **b**, TLC analysis of reaction products of KDRG catalyzed by recFlDeg. *Before*, before enzyme reaction; *After*, after enzyme reaction; *RP3*, fractions indicated by red lines in **a**.

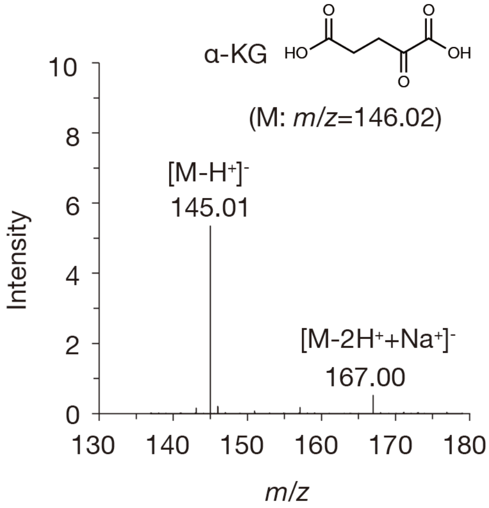

Supplementary Fig. 16. ESI-MS analysis of standard α-KG.

The negative ion ESI mass spectra of standard α-KG. Its structure is shown in the *inset*.

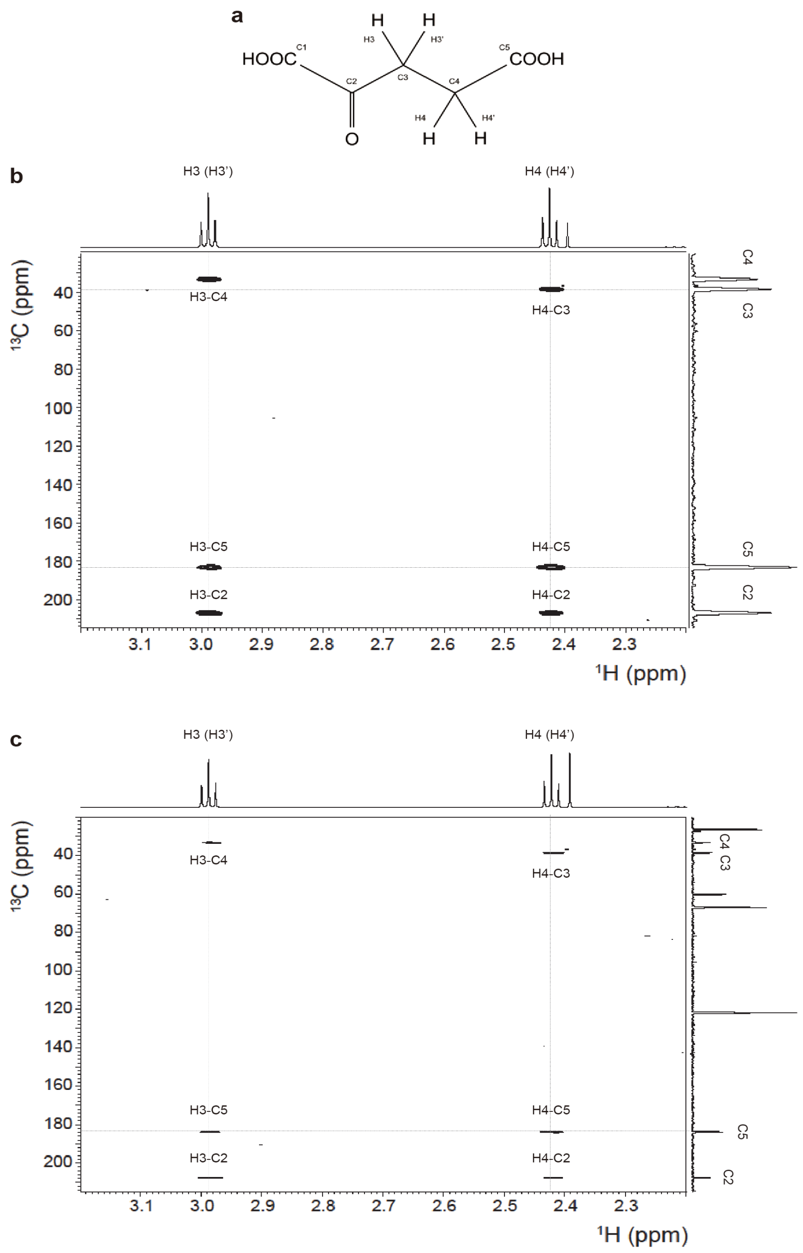

Supplementary Fig. 17. ^{1}H-^{13}C HMBC spectrum of standard α-KG and purified RP3.

a, Chemical structure of α-KG; **b** and **c**, ^{1}H-^{13}C HMBC spectrum of standard α-KG (**b**) and purified RP3 (**c**).

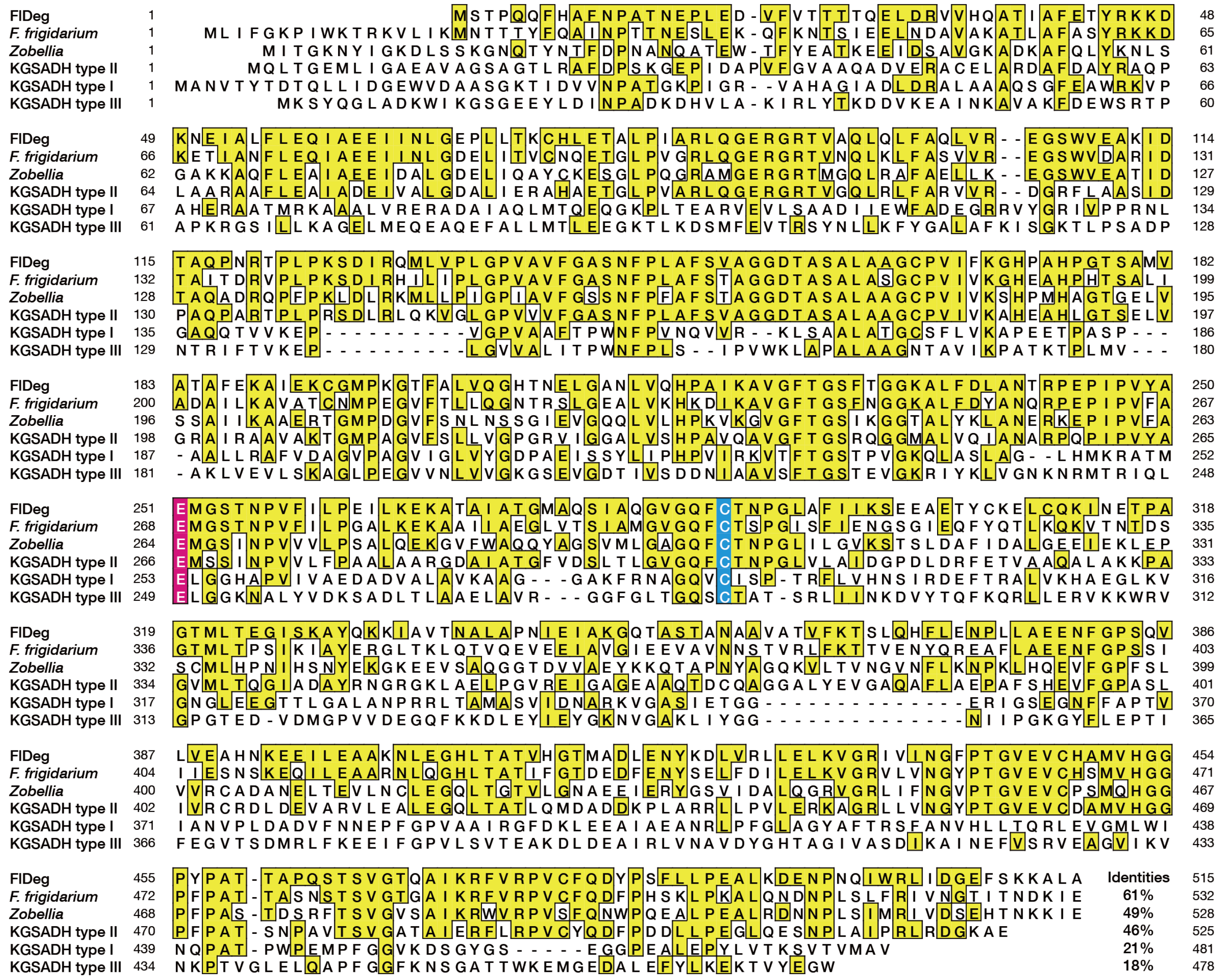

Supplementary Fig. 18. Comparison of amino acid sequences of FlDeg and its homologs.

FlDeg, *Flavobacterium sp.* strain UMI-01 FlDeg (in this study); *F. frigidarium*, KGSA dehydrogenase-like protein from *F. frigidarium* (GenBank accession number WP_026708994); *Zobellia*, KGSA dehydrogenase-like protein from *Z. galactanivorans* (GenBank accession number WP_013995335); *KGSADH type II*, KGSA dehydrogenase (Type II) from *Azospirillum brasilense* (GenBank accession number BAF33385); *KGSADH type I*, KGSA dehydrogenase (Type I) from *A. brasilense* (GenBank accession number Q1JUP4); *KGSADH type III*, KGSA dehydrogenase (Type III) from *Sulfolobus solfataricus* (GenBank accession number AAK43220). The amino acids highlighted in magenta or cyan are common nucleophiles or general bases in the ALDH superfamily.

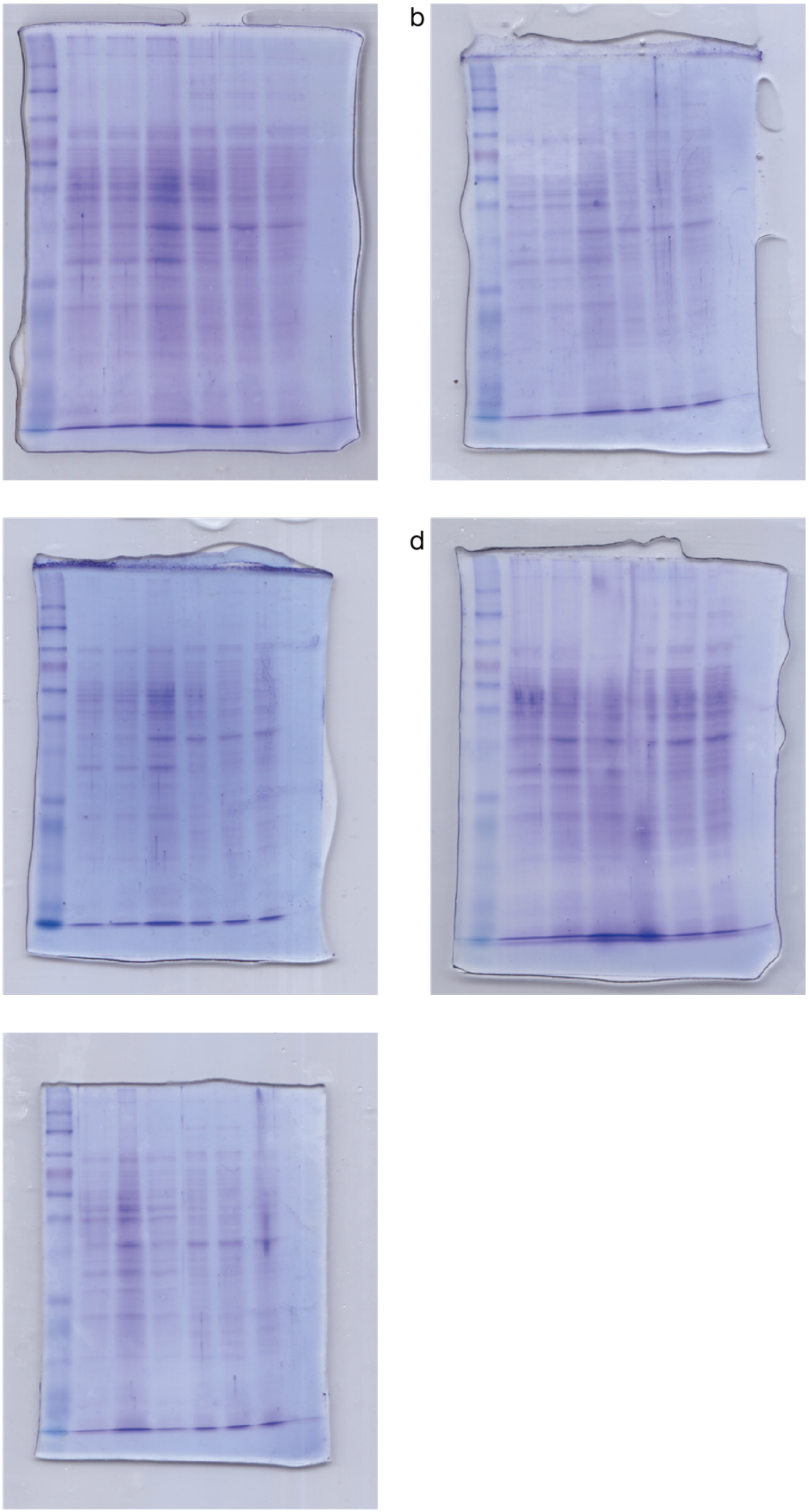

Supplementary Fig. 19. Uncropped and unedited CBB-stained gels in Western blot analysis.

a, **b**, **c**, **d**, and **e**, CBB-stained gels that were electrophoresed at the same time as the gels transferred for Western blot using anti-FlRed, -FlKin, -FlAld, -FlDet, and –FlDeg antibodies, respectively, in Fig. 5.

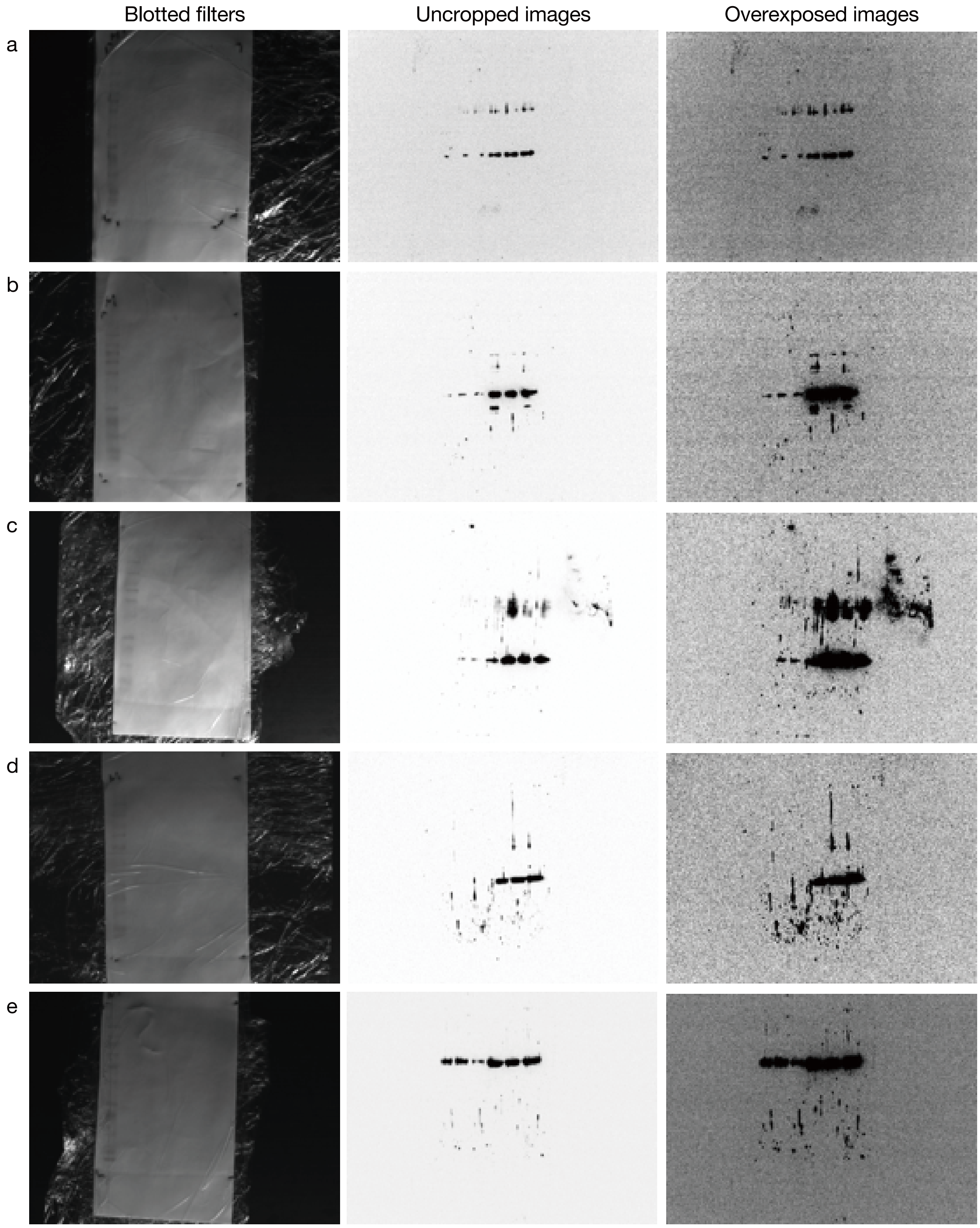

Supplementary Fig. 20. Blotted filters and uncropped images of Western blot analysis.

Blotted filters (*left*), uncropped images (*middle*), and overexposed images (*right*) of Western blot analysis with antibodies against FlRed, FlKin, FlAld, FlDet, and FlDeg.

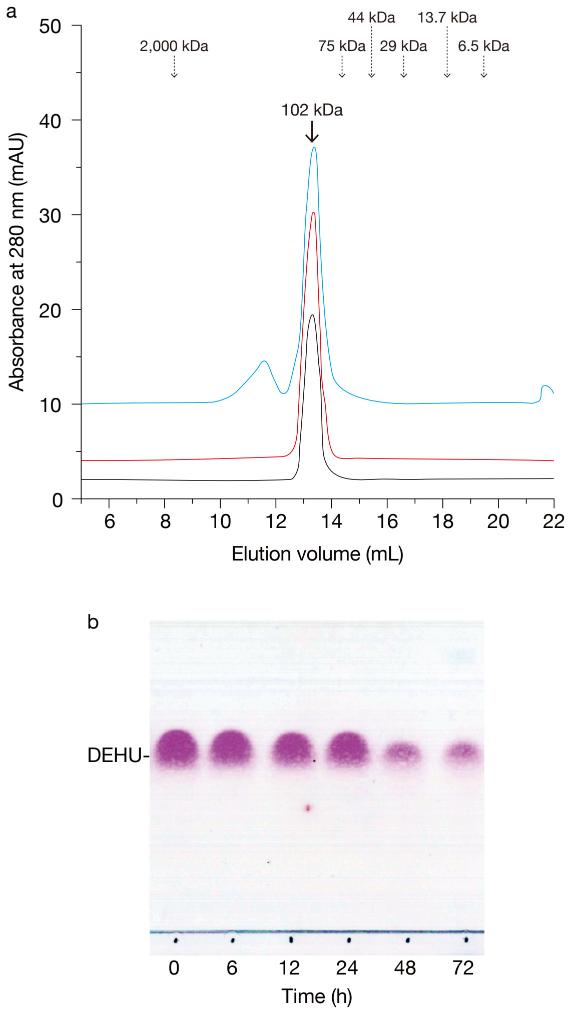

Supplementary Fig. 21. Gel filtration of native and recombinant FlReds (a) and the oxidation reaction of DEHU by the His-tagged FlRed (b).

a, native FlRed (black), recFlRed without His-tag (red), and the His-tagged FlRed (cyan) was applied to Superdex 200 10/200 GL gel filtration column (GE Healthcare, Chicago, IL, USA). Molecular weight was estimated using the HMW gel filtration calibration kit (GE Healthcare). Each elution position of the marker (blue dextran: 2,000 kDa, conalbumin: 75 kDa, ovalbumin: 40 kDa, carbonic anhydrase: 29 kDa, ribonuclease A: 13.7 kDa, and aprotinin: 6.5 kDa) is indicated by dotted arrows. *b*, TLC analysis (TBA staining method) of the reaction mixture containing 10 mM sodium phosphate (pH 7.4), 100 mM KCl, 1 mM DTT, 50 μg mL^{-1} His-tagged FlRed, and 25 mM DEHU with 5 mM NAD^{+} at 25°C.

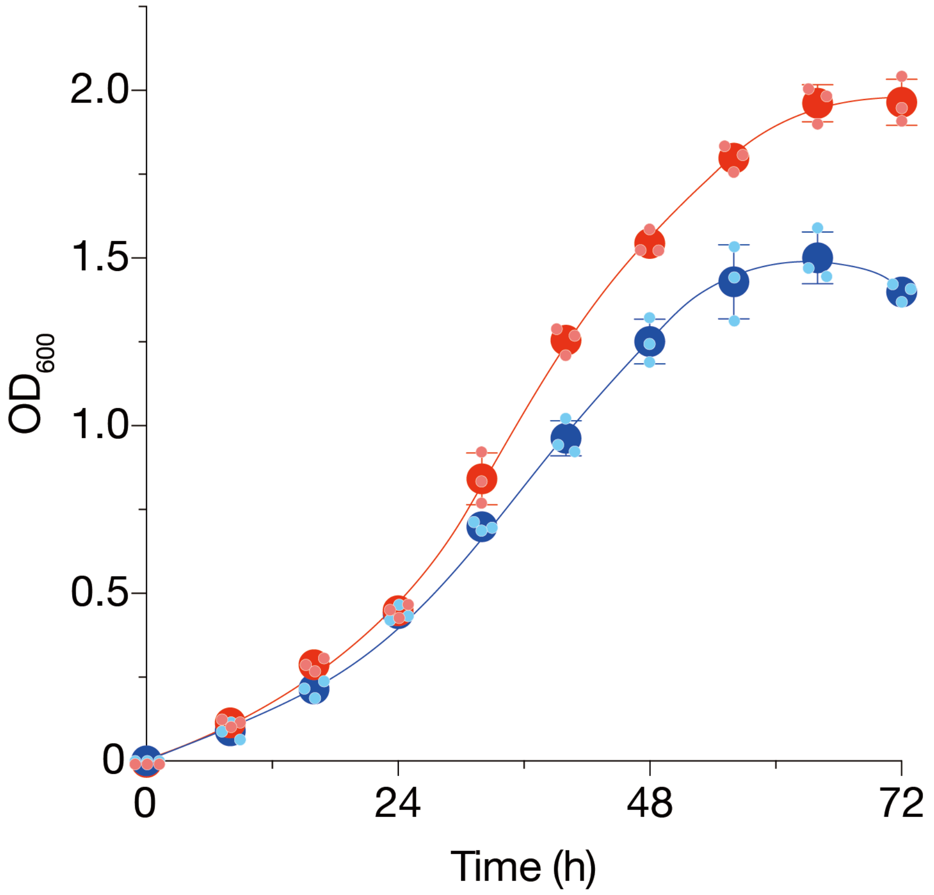

Supplementary Fig. 22. Growth curves of strain UMI-01.

Strain UMI-01 was grown at 25˚C in the alginate-minimum salt medium (1% (*w*/*v*) sodium alginate, 0.38% (*w*/*v*) Na_2HPO_4, 0.27% (*w*/*v*) KH_2PO_4, 0.036% (*w*/*v*) NH_4Cl, 0.02% (*w*/*v*) $MgCl_2$, and 0.1% (*v*/*v*) of trace element solution (0.97% (*w*/*v*) $FeCl_3$, 0.78% (*w*/*v*) $CaCl_2$, 0.02% (*w*/*v*) $CoCl_2$•$6H_2O$, $CuSO_4$•$5H_2O$, 0.01% (*w*/*v*) $NiCl_2$•$6H_2O$ and 0.01% $CrCl_3$•$6H_2O$ in 0.1 N HCl)) shown by red or the alginate-artificial seawater (calcium free) medium (1% (*w*/*v*) sodium alginate, 0.01% (*w*/*v*) K_2HPO_4 (*w*/*v*), 2.5% (*w*/*v*) NaCl, 0.1% (*w*/*v*) KCl, 0.05% (*w*/*v*) NH_4Cl, 0.5% (*w*/*v*) $MgSO_4$•$7H_2O$, and 0.002% (*w*/*v*) $FeSO_4$•$7H_2O$) shown by blue. The pH of each medium was adjusted to 7.5 with 1 N NaOH. Cultures were repeated three times and the data are shown as mean ± S.D.

Supplementary Table 1. Summary for purification of an enzyme with DEHU-oxidizing activity

Steps	Protein (mg)
Cell extract	451
TOYOPEARL Butyl-650M	28.1
TOYOPEARL SuperQ-650S	7.65
MonoQ 4.6/100 PE	2.76
Superdex 200 10/300 GL	1.32

Supplementary Table 2. Summary for purification of FlDet

Steps	Protein (mg)	Activity (U)	Specific activity (U mg^{-1})	Purification (fold)	Yield (%)
Cell extract	456	1.44	0.0032	1	100
TOYOPEARL Butyl-650M	5.13	1.03	0.200	62.5	72
MonoQ 4.6/100 PE	0.174	0.910	5.23	1634	63
Superdex 200 10/300 GL	0.033	0.280	8.48	2650	19

Supplementary Table 3. Coenzyme and substrate specificities of recFlDeg.

Coenzymes or substrates	Relative activity (%)
**Coenzyme**	
NAD^+	100±3.3
$NADP^+$	8.2±2.2
NADH	N.D.
NADPH	N.D.
**Substrate**	
**α-Keto acid**	
α-KGSA (C5)	100±3.3
DEHU (C6)	N.D.
**Aldehyde**	
Glutaraldehyde (C5)	8.5±0.44
o- Phthalaldehyde	1.2±0.13
Benzaldehyde	1.3±0.44
**Aldose**	
Glucose (C6)	N.D.
Galactose (C6)	N.D.
Arabinose (C5)	N.D.
Xylose (C5)	N.D.
**Uronic acid**	
Glucuronic acid (C6)	N.D.
**Deoxy sugar**	
2-deoxy-D-glucose (C6)	N.D.

N.D. means no activity was detected. For coenzyme assays, reaction was conducted in 10 mM potassium phosphate (pH 7.4), 100 mM KCl, 1 mM $MgCl_2$, 1 mM DTT, 10 µg mL^{-1} recFlDeg, 1 mM α-KGSA, and 0.5 mM coenzyme at 25°C. For substrate assays, reaction was conducted in 10 mM potassium phosphate (pH 7.4), 100 mM KCl, 1 mM $MgCl_2$, 1 mM DTT, 10 µg mL^{-1} recFlDeg, 0.5 mM NAD^+, and 1 mM substrate at 25°C. Relative activity at 100% was equivalent to 1.8 U mg^{-1}.

Supplementary Table 4. Primers used in this study.

Primers	Nucleotide sequence (5' to 3')	Use applications
FlRed-notag-F	AGGTAATACACCATGGGTAATTTAAATGAAAAAGTT	Expression of recFlRed.
FlRed-notag-R	CACCTCCACCGGATCCTTATATTCCTAAAGCTTGACCTCC	
FlDet-F	CCAGATTGGTTTACCAGTTTGGATTTCC	Cloning of FlDet gene.
FlDet-R	TAAGTGACCCATCACTCGCCTGATTACC	
FlDet-HisC-F	AGGTAATACACCATGACAACACTATCAGCTTCTACAA	Expression of recFlDet.
FlDet-HisC-R	CACCTCCACCGGATCCTTTATTTTTTTCCAGTTTTC	
FlDeg-F	GGTTAACCAGTTATGGTATTCCGGTGG	Cloning of FlDet gene.
FlDeg-R	GAGGTAATCAGGCGAGTGATGGGTCAC	
FlDeg-HisC-F	AGGTAATACACCATGAGCACTCCTCAACAATTTCATG	Expression of recFlDeg
FlDeg-HisC-R	CACCTCCACCGGATCCAGCGAGAGCTTTCTTTGAAAA	

Underlines indicate additional sequences for In-Fusion cloning.

Supplementary reference

1. Nishiyama, R., Inoue, A. & Ojima, T. Identification of 2-keto-3-deoxy-D-gluconate kinase and 2-keto-3-deoxy-D-phosphogluconate aldolase in an alginate-assimilating bacterium, *Flavobacterium* sp. strain UMI-01. *Mar. Drugs* **15,** 37 (2017).
