# Supplementary material for: An oxidative metabolic pathway of 4-deoxy-L-erythro-5-hexoseulose uronic acid (DEHU) from alginate in an alginate-assimilating bacterium
Source: Commun Biol. 2021 Nov 2;4:1254. doi: 10.1038/s42003-021-02786-8 (PMC8563752; doi:10.1038/s42003-021-02786-8)
Supplement: Supplementary file 3 — Description of Additional Supplementary Files. [file 42003_2021_2786_MOESM3_ESM.pdf]

Description of Additional Supplementary Files

File name: Supplementary Data 1

Description: The data for kinetic analysis in Table 1.

File name: Supplementary Data 2

Description: The analyzed MS and MS/MS data in Fig. 1e

File name: Supplementary Data 3

Description: The source data for the graphs in the main figures.

File name: Supplementary Data 4

Description: Uncropped and unedited SDS-PAGE gels in Fig.1c, 2b, and 2c.
