# Supplementary figures and images for: An oxidative metabolic pathway of 4-deoxy-L-erythro-5-hexoseulose uronic acid (DEHU) from alginate in an alginate-assimilating bacterium
Source: Commun Biol. 2021 Nov 2;4:1254. doi: 10.1038/s42003-021-02786-8 (PMC8563752; doi:10.1038/s42003-021-02786-8)

Fig. 1c

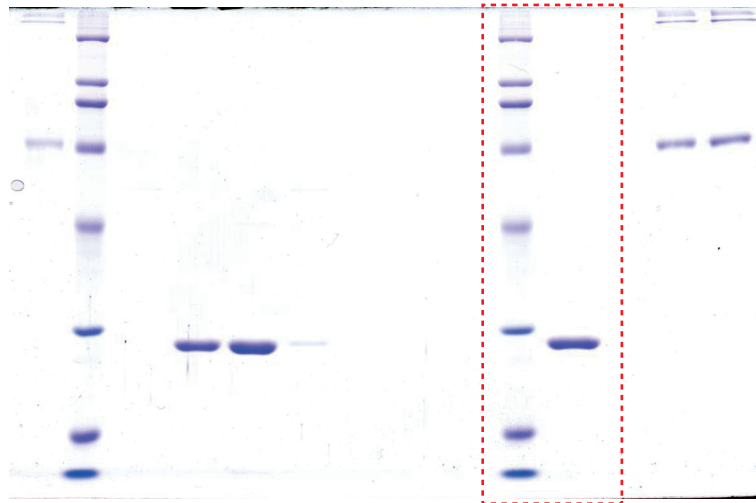

Fig. 2b

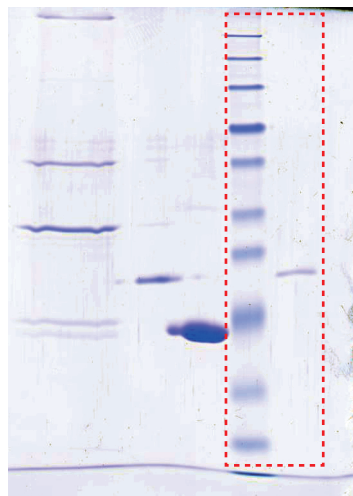

Fig. 2c

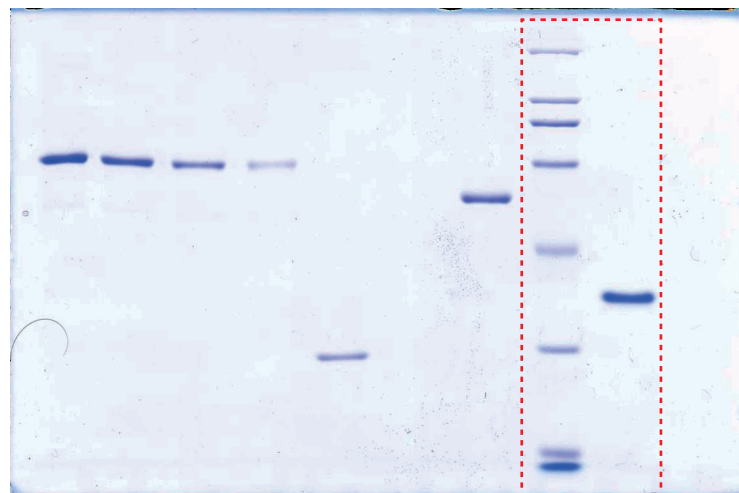

Supplement: Supplementary file 7 — Supplementary Data 4. [file 42003_2021_2786_MOESM7_ESM.pdf]
